# Cognitive Impairment in Rheumatoid Arthritis: The Role of Pain, Inflammation, and Multimorbidity in Neuropsychological Outcomes

**DOI:** 10.3390/biomedicines13071699

**Published:** 2025-07-11

**Authors:** Agnieszka Pigłowska-Juhnke, Maia Stanisławska-Kubiak, Piotr Kalmus, Marzena Waszczak-Jeka, Włodzimierz Samborski, Ewa Mojs

**Affiliations:** 1 Department of Clinical Psychology, Poznan University of Medical Sciences, 60-812 Poznań, Poland; 2 Institute of Health, WSG University, 85-229 Bydgoszcz, Poland; 3 Department of Rheumatology, Rehabilitation and Internal Medicine, Poznań University of Medical Sciences, 61-545 Poznań, Poland

**Keywords:** rheumatoid arthritis (RA), chronic pain, neuropsychological assessment, cognitive impairment, chronic inflammation

## Abstract

Rheumatoid arthritis (RA) is a chronic autoimmune disease that may affect the central nervous system, leading to cognitive impairment associated with chronic inflammation and pain. **Objective**: To assess the relationship between cognitive function, disease progression, pain intensity, and clinical parameters in patients with RA. **Materials and Methods**: This study included 62 RA patients, including individuals with comorbid conditions. Cognitive performance was assessed using the Automated Neuropsychological Assessment Metrics (ANAM) battery. Associations between cognitive function and pain intensity (VAS), inflammatory markers (ESR), number of disease flares, and surgical interventions were analyzed. **Results**: Patients with isolated RA demonstrated better performance in visuospatial memory and cognitive flexibility compared to those with comorbidities. Increased pain intensity and the number of disease flares were associated with impaired attention, memory, and psychomotor speed. **Conclusions**: Chronic pain and high disease activity in RA negatively impact cognitive functions. Routine neuropsychological assessment should be considered in the comprehensive clinical management of RA patients.

## 1. Introduction

Rheumatoid arthritis (RA) is a chronic and systemic autoimmune disease primarily characterized by progressive synovial inflammation, joint destruction, and functional disability. Traditionally conceptualized as a disease confined to the musculoskeletal system, accumulating evidence suggests that RA exerts far-reaching systemic effects, including the potential involvement of the central nervous system (CNS). Chronic inflammation, persistent pain, and complex neuroimmune interactions may contribute to subtle yet clinically significant cognitive impairments in this patient population [1].

Emerging research indicates that cognitive dysfunction in RA may be driven by multiple pathophysiological mechanisms. These include neuroinflammation induced by pro-inflammatory cytokines (e.g., IL-1β, IL-6, TNF-α) crossing the blood–brain barrier, secondary vascular changes increasing cerebrovascular risk, and psychosocial factors such as chronic fatigue, depression, anxiety, and sleep disturbances [2,3]. Additionally, pharmacological interventions commonly used in RA, including glucocorticoids and disease-modifying antirheumatic drugs (DMARDs)—both synthetic and biologic—may modulate cognitive processes, with potential neuroprotective or neurotoxic effects depending on dosage, duration, and individual susceptibility [4].

Cognitive symptoms in RA often present insidiously and may go unrecognized by clinicians and patients alike. Deficits in attention, working memory, processing speed, and executive functioning may be erroneously attributed to aging, chronic pain, or fatigue, yet they bear significant implications for patients’ quality of life, vocational productivity, and social engagement [1,5]. Furthermore, cognitive impairment has been associated with increased morbidity and mortality in chronic systemic diseases, underscoring the need for early detection and intervention.

Neuroimaging studies provide additional insight into the CNS’s involvement in RA. Structural MRI and functional imaging modalities such as SPECT have revealed hypoperfusion in the frontal and parietal lobes, increased white matter hyperintensities, and reductions in gray matter volume, particularly among patients with longstanding disease [6]. These findings align with hypotheses positing microangiopathic changes and disrupted neural connectivity between subcortical white matter and frontoparietal regions as potential contributors to cognitive deficits [7].

Despite these observations, neurocognitive assessment is not yet integrated into routine rheumatological care. This gap highlights an urgent need for a multidisciplinary approach that encompasses both rheumatological and neuropsychological expertise. Cognitive impairment in RA should be recognized as an important, though often overlooked, comorbidity warranting systematic evaluation and targeted intervention.

The present study aims to explore the relationship between cognitive function and disease-specific variables in patients with RA, including pain intensity, inflammatory markers, disease exacerbations, and surgical interventions. We further investigate the impact of comorbid conditions on neuropsychological outcomes. By employing a comprehensive battery of computerized cognitive assessments (ANAM), we offer a nuanced perspective on the cognitive challenges faced by patients with RA. Our findings underscore the imperative for interdisciplinary collaboration in optimizing clinical outcomes and quality of life for this patient population.

## 2. Materials and Methods

### 2.1. Study Objective

The objective of this study was to evaluate the relationship between cognitive function and clinical as well as sociodemographic parameters in patients with rheumatoid arthritis (RA). Specifically, we investigated the impact of disease diagnosis, pain severity, and disease course—measured by erythrocyte sedimentation rate (ESR), frequency of disease exacerbations, and the number of joint surgeries—on selected cognitive domains.

### 2.2. Study Design and Setting

This study was conducted at a health resort rehabilitation hospital as part of a standard inpatient spa treatment. The study protocol received ethical approval from the Bioethics Committee of the Karol Marcinkowski University of Medical Sciences in Poznań.

### 2.3. Participants

A total of 62 patients with a confirmed RA diagnosis were enrolled in this study. The inclusion criteria comprised age ≥ 18 years, having completed at least primary-level education, and the provision of written informed consent. Exclusion criteria included medical conditions that could potentially influence cognitive performance (e.g., significant neurological or psychiatric disorders) and substance use disorders.

Patients with RA were stratified into two subgroups: (1) those with isolated RA and (2) those with RA comorbid with other chronic somatic diseases.

### 2.4. Assessment Tools

This study employed a set of standardized assessment tools, including the following: Sociodemographic Questionnaire: Collected data on age, sex, marital status, education level, place of residence, financial status, and clinical information (disease duration, medications, frequency of disease exacerbations, number of joint surgeries, highest historical ESR values, and presence of comorbidities). Visual Analog Scale (VAS): Assessed pain intensity. Beck Depression Inventory (BDI): Used as a screening tool for mood disturbances. Medical Record Review: Included diagnostic history and current treatment regimen. Rheumatologist-Assessed Functional Health Status: Evaluated clinical disease activity. Erythrocyte Sedimentation Rate (ESR): Used as an inflammatory activity marker.

### 2.5. Neuropsychological Assessment

Cognitive functions were evaluated using the Automated Neuropsychological Assessment Metrics (ANAM), version GNS. This battery consists of 18 tests assessing problem-solving abilities, cognitive flexibility, response inhibition, working and visuospatial memory, processing speed, and attention. All tests were administered using an HP computer running Windows 7, meeting the ANAM system specifications.

The neuropsychological assessment lasted between 75 and 100 min. Upon completion, patients received an individualized discussion of their test results, recommendations for further management, and access to psychoeducational materials and cognitive training exercises. In two cases, referrals were made to a psychogeriatric clinic, and three participants were advised to seek psychotherapy or psychiatric consultation.

Based on prior studies, the sensitivity and specificity of the ANAM battery for detecting cognitive impairment were reported as 81% and 89.1%, respectively [8]. The test battery demonstrates the highest diagnostic accuracy in measuring attention, processing speed, and working memory [9,10].

### 2.6. Standardization and Data Analysis

The assessment procedure was standardized, with all tests conducted under identical environmental conditions at predetermined times by the same trained personnel. Data from the ANAM system were exported to a Microsoft Excel database and subsequently analyzed using IBM SPSS Statistics 24.

The normality of the data distribution was assessed using the Kolmogorov–Smirnov test. Given that most variables demonstrated nonparametric distributions, nonparametric statistical methods were employed. The Mann–Whitney U test was used for comparisons between independent groups, while Spearman’s rank correlation coefficient (r_s_) was applied to evaluate associations between variables. Additionally, to determine effect sizes, Glass’s biserial correlation coefficient was computed.

Descriptive statistics were reported using central tendency and variability measures, including mean, standard deviation, median, mode, and range (minimum–maximum values).

All analyses were conducted with a predefined significance level of *p* < 0.05, with a stricter threshold of *p* < 0.01 applied in more rigorous statistical assessments. Results were deemed statistically significant if the p-values did not exceed the predefined thresholds. Confidence intervals were set at 95%.

## 3. Results

### 3.1. Participant Characteristics

The study cohort comprised 62 patients diagnosed with rheumatoid arthritis (RA), with a predominant representation of women (90.3%). The mean age of participants was 61.4 years (SD = 9.7), ranging from 39 to 81 years. The majority of patients resided in urban areas, and their educational attainment was primarily at the secondary level (46.8%), followed by vocational and higher education (both 24.2%).

More than half of the participants (56.5%) had experienced at least four disease exacerbations, whereas 59.7% had no history of joint surgery. The mean disease duration since diagnosis was 15.6 years. In terms of inflammatory markers, the highest historical erythrocyte sedimentation rate (ESR) recorded averaged 76 mm/h, while the current ESR values at the time of assessment averaged 24.7 mm/h.

### 3.2. Pain Intensity and Mood Assessment

The mean current pain intensity, as measured by the Visual Analog Scale (VAS), was 3.2 (SD = 1.8), while the highest pain levels reported in the past averaged 8 points (SD = 2.1).

In the mood screening using the Beck Depression Inventory (BDI), the majority of participants (74.2%) did not exhibit depressive symptoms. However, 21% met the criteria for mild depression, and 4.8% were classified as having moderate depression.

### 3.3. Neuropsychological Performance

Cognitive function was systematically assessed using the Automated Neuropsychological Assessment Metrics (ANAM) test battery, allowing for a detailed evaluation of key cognitive domains, including working memory, attention, cognitive flexibility, response inhibition, and processing speed.

### 3.4. Functional Performance Metrics

Among the RA patients, 25% exhibited significant physical impairment, whereas 38% retained full functional capacity. An additional 38% reported the sufficient ability to perform activities of daily living, despite the presence of joint pain.

The mean disease duration was 15 years (range: 2–44 years). The current pain intensity, as measured on the VAS, was low (mean: 3.2), whereas the highest pain experienced reached an average of 8 points. Inflammatory activity, assessed via ESR, showed a maximum recorded value of 100 mm/h, while the mean current ESR at the time of the study was 24.7 mm/h.

### 3.5. Disease Severity and Treatment

More than half of the patients (56.5%) experienced frequent exacerbations (more than four per year), and 10% reported a persistently active disease course. A history of joint surgery was documented in 40% of participants, with most undergoing one to two procedures.

Regarding pharmacological management, the following were determined:

- 71% of patients were treated with methotrexate;
- 56% received corticosteroids;
- 40% were on nonsteroidal anti-inflammatory drugs (NSAIDs);
- 3% were undergoing biologic therapy.

### 3.6. Cognitive Function in RA Patients

The prevalence of depressive symptoms (based on BDI) was 26%, with 21% classified as mild and 5% as moderate depression.

A comparative analysis of cognitive performance in RA patients versus those with RA and coexisting chronic conditions is summarized in Table 1.

The results presented in Table 1 indicate that individuals with rheumatoid arthritis (RA) differ from those with RA and comorbid conditions in the following cognitive domains: Immediate Visuospatial Memory (M), Immediate Visuospatial Memory (E), Visuospatial Orientation (M), Visuospatial Orientation (E), Cognitive Flexibility (P), and Cognitive Flexibility (E).

Individuals with RA, compared to those with RA and comorbidities, demonstrated superior performance in Immediate Visuospatial Memory (M), Immediate Visuospatial Memory (E), Visuospatial Orientation (M), Visuospatial Orientation (E), Cognitive Flexibility (P), and Cognitive Flexibility (E).

The findings presented in Table 2 indicate that age was significantly, though weakly, positively correlated with Simple Reaction Time (M), while also showing a significant, weak, and negative correlation with Simple Reaction Time (E), Reaction Time in Information Processing (P), Mental Arithmetic (P), Mental Arithmetic (W), Immediate Visuospatial Memory (P), and Immediate Visuospatial Memory (W). Furthermore, current pain intensity was found to be significantly, weakly, and positively correlated with Immediate Visuospatial Memory (M), Inhibition (P), Inhibition (H), Spatial Orientation (M), and Spatial Orientation (P). At the same time, significant weak and negative correlations were observed with Immediate Visuospatial Memory (E) and (P), Inhibition (ER), and Sustained Attention (H). The maximum pain intensity experienced was significantly, weakly, and positively correlated with Learning (P), Task Switching (P), and Psychomotor Speed of the Right Hand (M), while it was significantly, weakly, and negatively correlated with Immediate Visuospatial Memory (P) and Psychomotor Speed of the Right Hand (N).

The number of disease exacerbations was found to be significantly, weakly, and negatively correlated with Recall (P), Immediate Visuospatial Memory (E), and Visuospatial Orientation 2 (P). The number of past surgeries was significantly, weakly, and positively correlated with Learning (P).

Pain Intensity and Cognitive Function in RA Patients: In the analyzed group of RA patients, it was observed that higher levels of currently experienced pain were associated with lower performance in immediate visuospatial memory tasks. Specifically, individuals experiencing greater pain on the day of the assessment provided fewer correct responses and exhibited prolonged reaction times. Elevated pain levels also had a negative impact on spatial orientation (measured as the mean response time for correct answers) and sustained attention (measured by the number of correct hits).

Additionally, chronic pain associated with RA was linked to a reduced number of correct responses in immediate visuospatial memory tasks, as well as decreased psychomotor performance of the right hand (measured by the number of key presses).

Inflammatory Markers and Cognitive Function: The erythrocyte sedimentation rate (ESR) did not demonstrate a clear association with the functional status of patients, as assessed by the Gofton scale. However, the number of disease exacerbations had a significant impact on performance declines in immediate visuospatial memory, visuospatial orientation, and delayed recall tasks. These findings suggest that frequent disease relapses may contribute to impairments in information storage and retrieval abilities, irrespective of stimulus modality. This phenomenon, however, warrants further in-depth investigation.

Age and Cognitive Processing Efficiency: Finally, the results also demonstrated that older age was significantly, though weakly, correlated with slower reaction times in tasks requiring information processing, reduced efficiency in mental arithmetic tasks, and impaired immediate visuospatial memory.

Additional statistical analyses included multiple linear regressions and cluster analyses (Table 3). The regression models incorporated age, duration of RA, and steroid use as predictors of cognitive performance. Regression analyses were conducted on the data from 59 participants. Three participants were excluded due to missing values regarding steroid use. These analyses revealed that age was a consistent and significant predictor for several cognitive outcomes (learning, spatial memory, cognitive flexibility). Steroid use was positively associated with working memory performance, but this association did not reach statistical significance (*p* = 0.072). Given the highly unbalanced group sizes (51 users vs. 8 non-users), this trend should be interpreted with caution, as it may reflect sampling bias rather than suggest a true pharmacological effect. Disease duration was not significantly related to cognitive outcomes.

For reaction time, the regression model indicated a trend toward significance for the predictor age (*p* = 0.095), suggesting a potential but not statistically confirmed relationship that may warrant further investigation in larger samples.

Cluster analysis further identified subgroups of RA patients with distinct cognitive profiles, highlighting the heterogeneity of cognitive function in this population.

## 4. Discussion

It is increasingly recognized that assessing cognitive function in patients with autoimmune diseases, including rheumatoid arthritis (RA), is of significant importance. This heightened focus has emerged in parallel with growing evidence of a bidirectional communication axis between the immune system and the central nervous system (CNS) [10,11,12]. Chronic systemic inflammation, the hallmark of RA, not only damages joint structures but may also disrupt CNS function, thereby contributing to subtle yet clinically relevant cognitive deficits [4,11].

In the present study, cognitive impairments in RA patients—though often imperceptible to others—significantly compromised quality of life by hindering daily activities, professional performance, and social engagement. The subtle cognitive deficits we observed most commonly affected working memory, executive function, attention, and visuospatial information processing. Notably, our findings are consistent with earlier reports indicating that 30–71% of RA patients may experience cognitive dysfunction [13], underscoring that neuropsychological changes are a prevalent yet under-recognized component of RA.

We paid particular attention to the relationships between pain intensity, inflammatory markers, disease severity, and neuropsychological performance. Our analysis revealed that patients experiencing greater pain on the day of cognitive evaluation performed worse on tests of immediate visuospatial memory, spatial orientation, and sustained attention. Patients reporting higher pain levels produced fewer correct responses and exhibited longer reaction times on tasks requiring rapid information processing. This pattern is consistent with prior evidence that chronic pain can significantly disrupt cognitive function, likely through effects on neural networks subserving both pain perception and cognitive processing [14,15].

Prior studies offer insight into the neurobiological underpinnings of the cognitive deficits seen in RA. For instance, Bartolini et al. (2002) have suggested that impairments in visuospatial abilities and executive functions may stem from RA-related white matter damage and cerebral microangiopathy [7]. Supporting this notion, Shin et al. (2012) reported deficits in spatial processing and attention among RA patients. Consistent with these findings, we observed that deficits in spatial orientation and visuospatial memory were especially pronounced in our patients who experienced frequent disease exacerbations [5].

Furthermore, we found that a higher number of disease flares was significantly associated with greater impairment in immediate visuospatial memory and recall. This observation suggests that active disease phases and recurrent inflammatory relapses may exert a deleterious effect on cognitive functions. Such an interpretation aligns with reports implicating pro-inflammatory cytokines (e.g., IL-6 and TNF-α) in the modulation of cognitive processes [10,11], raising the possibility that heightened cytokine activity during flares contributes to neurocognitive changes in RA.

Interestingly, the inflammatory marker erythrocyte sedimentation rate (ESR) showed no clear association with cognitive performance measures, indicating that conventional laboratory indices of inflammation may not fully capture the extent of CNS involvement in RA [11]. Likewise, the number of joint surgeries a patient had undergone did not correlate with neuropsychological test outcomes. This lack of association might reflect the complexities of surgical decision-making in RA and the adoption of modern anesthetic techniques designed to mitigate the neurotoxicity risk [16].

Importantly, an age-related effect on cognition was evident in our cohort. Older RA patients tended to exhibit somewhat significantly prolonged reaction times on information-processing tasks, along with modest reductions in performance on tasks involving mental arithmetic and memory. Although these age-associated cognitive declines were relatively subtle, they underscored the need to account for age as a factor when interpreting cognitive test results in RA populations [10,11].

The findings of our regression analyses indicate a significant association between certain clinical factors and cognitive functioning in patients with rheumatoid arthritis (RA). Specifically, age emerged as the most robust and consistent predictor of declines in learning, spatial memory, and cognitive flexibility. This aligns with prior research demonstrating that aging is a key factor in cognitive decline among RA patients [11].

Additionally, the use of glucocorticoids showed a positive, though not statistically significant, association with working memory performance. While some studies suggest that glucocorticoids may have neuroprotective effects in specific contexts [17], their long-term use is also linked to potential adverse neuropsychological outcomes. Therefore, the observed association warrants further investigation to elucidate the underlying mechanisms and to balance the benefits and risks of glucocorticoid therapy in RA patients [2,17].

Overall, these findings underscore the importance of considering both age and medication use when assessing cognitive function in RA patients. Future studies should aim to explore these relationships further, ideally incorporating additional inflammatory markers and larger, more diverse cohorts to enhance generalizability.

### Innovation and Novelty of the Study

The present study offers notable contributions to the literature on cognitive function in rheumatoid arthritis (RA) through both its methodological rigor and its contextual positioning within existing research. One methodological strength lies in the use of the Automated Neuropsychological Assessment Metrics (ANAM), a standardized computerized test battery that allowed us to comprehensively assess a wide range of cognitive domains, including attention, memory, processing speed, and executive functions. Additionally, we stratified participants into two groups—RA patients with and without comorbidities—which enabled a more precise evaluation of the impact of multimorbidity on cognitive outcomes and reduced potential confounding factors.

We also acknowledge the importance of situating our findings within the broader context of the existing literature. Several prior studies, including those by Meade et al. [4], Pankowski et al. [11], and Shin et al. [5], have reported attention and memory deficits in RA patients. However, these studies did not examine the concurrent role of pain intensity or disease flares in cognitive outcomes. By contrast, our results highlight that both factors may contribute meaningfully to neuropsychological performance.

Our observation that pain intensity on the day of testing negatively influenced executive function and visuospatial memory aligns with previous findings by Moriarty et al. [14] and Berryman et al. [15]. Notably, our study expands upon these earlier insights by using both correlational and regression analyses to quantify these associations, providing a more nuanced picture of how pain modulates cognition in RA.

We also address the unclear relationship between ESR and cognitive function, echoing the conclusions of Liu et al. [16] and Meade et al. [4], who similarly found that traditional markers of systemic inflammation may not reliably reflect the extent of central nervous system involvement in RA.

Lastly, we discussed the potential effects of glucocorticoid use on working memory, referencing Hanly et al. [18] and Fazel et al. [17]. While our regression model suggested a positive association, we interpreted this result with caution due to the notable imbalance in subgroup sizes (51 users vs. 8 non-users), which may have limited the generalizability of this finding.

## 5. Conclusions

Patients with isolated RA demonstrated superior performance in immediate visuospatial memory, spatial orientation, and cognitive flexibility compared to those with RA and comorbid chronic diseases. These findings underscore the significant impact of multimorbidity on cognitive function in this population.

It should be noted that most patients in this study were treated with conventional therapies, as biologic agents are still not widely accessible in many countries. This treatment landscape is representative of real-world practices in numerous regions, which may influence generalizability.

Current pain intensity negatively influenced executive function, particularly response inhibition in neuropsychological tasks. This finding may have clinical relevance for daily functioning and decision-making processes in RA patients.

Higher levels of pain at the time of testing were associated with poorer visuospatial memory, longer reaction times in spatial orientation tasks, and reduced sustained attention. These results support the hypothesis that chronic pain negatively affects sensory integration and cognitive processing efficiency.

Pain severity in RA was also linked to decreased psychomotor performance, particularly in fine motor movements, as assessed by the number of key presses in cognitive tasks.

An increased number of disease exacerbations was associated with greater impairment in visuospatial memory, visuospatial orientation, and delayed recall. These findings highlight the importance of disease activity control not only for joint health but also for cognitive function.

The number of RA-related surgeries showed a weak but significant positive correlation with learning performance on visuospatial material. This may suggest that patients undergoing surgical interventions demonstrated greater cognitive adaptability postoperatively, though this relationship requires further investigation.

### Study Limitations

This study has certain limitations that should be acknowledged:

Lack of a healthy control group: Comparisons were made between patients with isolated RA and those with RA and comorbidities, which limited the generalizability of the findings.

Potential limitations of assessment tools: Some survey questions, particularly those related to pain intensity measured using the VAS scale, may have introduced subjective bias into data collection. The self-reported nature of pain and potential difficulties in recalling health-related information could have influenced the reliability of responses.

Use of the Beck Depression Inventory (BDI): Although widely used, the BDI does not fully align with the latest DSM-5 and ICD-11 diagnostic criteria, potentially limiting its diagnostic accuracy in assessing depressive symptoms.

Absence of verbal cognitive assessments: The ANAM battery primarily evaluates non-verbal cognitive functions, omitting the assessment of verbal memory and language-related cognitive domains, which could provide a more comprehensive evaluation of cognitive impairment in RA.

A key limitation of this study is the use of ESR as the sole inflammatory marker; future research should incorporate more specific biomarkers such as CRP, IL-6, or TNF-alpha to better capture systemic inflammation in RA.

A limitation of this study is the absence of additional inflammatory markers, such as CRP, which could have provided more comprehensive insights into systemic inflammation. Although the sample size was relatively small, it compared favorably to similar studies and offered valuable insights into cognitive functioning in RA.

A limitation of this study is the absence of direct comparisons with ANAM normative data, which could provide a valuable context for interpreting cognitive performance in RA patients.

## Figures and Tables

**Table 1:** Cognitive function test results in patients with rheumatoid arthritis (RA) and RA with comorbidities—analysis using the Mann–Whitney U test.

Variable	Disease			
	RA (*n* = 29)	RA with Comorbidities (*n* = 33)	Mann–Whitney U	*p*
	*M*	*M*		
Simple Reaction Time (M)	29.36	33.38	−0.87	0.382
Simple Reaction Time (E)	33.64	29.62	−0.87	0.382
Simple Reaction Time 2 (M)	35.45	28.03	−1.62	0.106
Simple Reaction Time 2 (E)	27.55	34.97	−1.62	0.106
Learning (M)	32.17	30.91	−0.28	0.783
Learning (P)	33.17	30.03	−0.70	0.487
Learning (E)	30.86	32.06	−0.26	0.794
Reaction Time in Information Processing (M)	28.03	34.55	−1.42	0.156
Reaction Time in Information Processing (P)	32.97	30.21	−0.65	0.517
Reaction Time in Information Processing (E)	35.03	28.39	−1.45	0.148
Task Switching (M)	28.91	33.77	−1.06	0.290
Task Switching (P)	32.16	30.92	−0.28	0.781
Task Switching (E)	34.02	29.29	−1.03	0.303
Mathematical Operations (M)	30.28	32.58	−0.50	0.616
Mathematical Operations (P)	32.50	30.62	−0.42	0.673
Mathematical Operations (E)	32.93	30.24	−0.59	0.558
Working Memory (M)	32.50	30.62	−0.41	0.682
Working Memory (P)	29.97	32.85	−0.63	0.526
Working Memory (E)	30.64	32.26	−0.35	0.724
Immediate Visuospatial Memory (M)	26.45	35.94	−2.07 *	0.039
Immediate Visuospatial Memory (P)	35.45	28.03	−1.63	0.104
Immediate Visuospatial Memory (E)	36.38	27.21	−2.00 *	0.046
Recall (M)	31.41	31.58	−0.04	0.972
Recall (P)	31.47	31.53	−0.01	0.989
Recall (E)	30.97	31.97	−0.22	0.827
Inhibition (M)	32.24	30.85	−−0.30	0.162
Inhibition (P)	30.10	32.73	−0.57	0.567
Inhibition (H)	28.07	34.52	−1.42	0.155
Inhibition (ER)	30.47	32.41	−0.43	0.669
Spatial Orientation (M)	29.97	32.85	−0.63	0.530
Spatial Orientation (P)	32.86	30.30	−0.57	0.565
Spatial Orientation (E)	34.17	29.15	−1.09	0.274
Visuospatial Orientation (M)	26.31	36.06	−2.12 *	0.034
Visuospatial Orientation (P)	31.57	31.44	−0.03	0.997
Visuospatial Orientation (E)	37.12	26.56	−2.30 *	0.021
Problem Solving (M)	30.36	32.50	−0.47	0.642
Problem Solving (MR)	28.14	34.45	−1.38	0.168
Psychomotor Speed Right Hand (M)	27.57	34.95	−1.61	0.108
Psychomotor Speed Right Hand (N)	35.33	28.14	−1.57	0.117
Psychomotor Speed Left Hand (M)	29.09	33.62	−0.99	0.323
Psychomotor Speed Left Hand (N)	33.78	29.50	−0.93	0.352
Sustained Attention (M)	28.64	34.02	−1.17	0.242
Sustained Attention (P)	33.79	29.48	−1.10	0.271
Sustained Attention (H)	31.88	31.17	−0.23	0.882
Sustained Attention (ER)	29.43	33.32	−1.19	0.233
Visuospatial Orientation 2 (M)	27.52	35.00	−1.63	0.103
Visuospatial Orientation 2 (P)	35.34	28.12	−1.58	0.115
Visuospatial Orientation 2 (E)	36.00	27.55	−1.84	0.066
Cognitive Flexibility (M)	28.97	33.73	−1.04	0.300
Cognitive Flexibility (P)	37.64	26.11	−2.52 *	0.012
Cognitive Flexibility (E)	36.52	27.09	−2.05 *	0.040

* *p* < 0.05; (M)—mean reaction time for correct responses; (P)—percentage of correct responses; (E)—efficiency; (H)—hits; (ER)—errors; (R)—movement ratio; (N)—number of key presses. Source: Own elaboration/Author’s own work.

**Table 2:** Age, current and maximum pain levels, maximum ESR, disease exacerbations, and past surgeries in relation to cognitive processes—Spearman’s rank correlation coefficients (r_s_).

	Age	Current Pain Level	Maximum Pain Level	Maximum ESR (Erythrocyte Sedimentation Rate)	Number of Disease Exacerbations	Number of Surgeries
Simple Reaction Time (M)	0.264 *	0.048	0.014	0.137	−0.026	−0.002
Simple Reaction Time (E)	−0.264 *	−0.048	−0.014	−0.137	0.026	0.002
Simple Reaction Time 2 (M)	−0.062	−0.126	−0.017	0.037	0.072	0.041
Simple Reaction Time 2 (E)	0.062	0.126	0.017	−0.037	−0.072	−0.041
Learning (M)	−0.087	0.171	0.189	0.032	0.106	0.219
Learning (P)	0.136	0.134	0.243 *	0.259 *	−0.044	0.288 *
Learning (E)	0.113	−0.156	−0.143	−0.014	−0.105	−0.176
Reaction Time in Information Processing (M)	−0.021	0.192	−0.062	0.078	0.075	−0.047
Reaction Time in Information Processing (P)	−0.285 *	−0.057	0.085	−0.038	−0.090	0.103
Reaction Time in Information Processing (E)	−0.051	−0.173	0.042	−0.078	−0.091	0.040
Task Switching (M)	0.122	0.039	0.095	0.187	−0.135	0.143
Task Switching (P)	0.053	0.028	0.215 *	0.099	0.148	0.155
Task Switching (E)	−0.072	−0.024	0.009	−0.154	0.189	−0.067
Mathematical Operations (M)	0.108	−0.008	0.087	−0.132	−0.118	0.086
Mathematical Operations (P)	−0.341 **	0.142	−0.002	0.320 *	0.183	−0.027
Mathematical Operations (E)	−0.222 *	−0.024	−0.071	0.261 *	0.166	−0.076
Working Memory (M)	−0.041	0.056	0.149	0.169	−0.175	0.046
Working Memory (P)	0.132	0.020	−0.028	−0.184	−0.001	0.013
Working Memory (E)	0.158	0.061	−0.111	−0.050	0.170	−0.021
Immediate Visuospatial Memory (M)	0.064	0.233 *	0.030	0.204	0.201	0.199
Immediate Visuospatial Memory (P)	−0.431 **	−0.258 *	−0.218 *	−0.112	−0.129	0.040
Immediate Visuospatial Memory (E)	−0.290 *	−0.353 **	−0.128	−0.142	−0.235 *	−0.112
Recall (M)	−0.161	0.092	−0.103	−0.056	0.014	0.055
Recall (P)	0.060	0.039	−0.144	−0.248 *	−0.249 *	−0.043
Recall (E)	0.162	−0.050	−0.012	−0.091	−0.181	−0.058
Inhibition (M)	−0.046	−0.066	0.042	−0.022	0.019	−0.048
Inhibition (P)	−0.166	0.232 *	0.045	0.121	0.038	0.073
Inhibition (H)	−0.149	0.224 *	0.000	0.199	0.013	0.039
Inhibition (ER)	0.199	−0.312 **	−0.172	0.006	−0.119	−0.156
Spatial Orientation (M)	−0.026	0.220 *	0.197	0.240 *	0.106	0.109
Spatial Orientation (P)	0.002	0.277 *	0.099	0.134	0.072	0.037
Spatial Orientation (E)	0.040	−0.115	−0.106	−0.166	−0.018	−0.039
Visuospatial Orientation (M)	0.089	0.119	0.081	0.177	0.105	0.131
Visuospatial Orientation (P)	−0.087	0.121	0.003	−0.155	−0.085	0.089
Visuospatial Orientation (E)	−0.068	−0.056	−0.064	−0.205	−0.138	−0.023
Problem Solving (M)	0.052	−0.072	0.025	0.165	−0.167	0.038
Problem Solving (MR)	0.141	−0.052	0.072	0.077	0.006	0.161
Psychomotor Speed Right Hand (M)	−0.047	0.163	0.274 *	0.052	−0.085	0.201
Psychomotor Speed Right Hand (N)	0.044	−0.162	−0.280 *	−0.049	0.084	−0.203
Psychomotor Speed Left Hand (M)	0.017	0.169	0.190	0.038	−0.036	0.174
Psychomotor Speed Left Hand (N)	−0.018	−0.177	−0.200	−0.023	0.038	−0.171
Sustained Attention (M)	−0.020	−0.014	0.013	−0.071	−0.163	−0.011
Sustained Attention (P)	0.137	−0.203	−0.026	0.085	−0.212	0.153
Sustained Attention (H)	−0.005	−0.231 *	−0.012	0.001	−0.155	0.152
Sustained Attention (ER)	−0.065	0.041	−0.031	−0.097	0.163	−0.123
Visuospatial Orientation 2 (M)	−0.016	0.105	0.139	0.097	0.037	0.144
Visuospatial Orientation 2 (P)	−0.010	−0.085	0.127	−0.032	−0.267 *	−0.084
Visuospatial Orientation 2 (E)	0.018	−0.106	−0.038	−0.054	−0.149	−0.131
Cognitive Flexibility (M)	−0.003	0.096	0.210	−0.002	0.172	0.157
Cognitive Flexibility (P)	−0.059	−0.136	0.124	−0.036	−0.052	−0.161
Cognitive Flexibility (E)	0.016	−0.139	−0.130	−0.059	−0.142	−0.216

* *p* < 0.05, ** *p* < 0.01; (M)—mean reaction time for correct responses; (P)—percentage of correct responses; (E)—efficiency; (H)—hits; (ER)—errors; (R)—movement ratio; (N)—number of key presses. Source: Own elaboration/Author’s own work.

**Table 3:** Summary of linear regression analyses (cognitive variables as dependent outcomes).

Cognitive Variable	R^2^	Age (β, *p*)	Disease Duration (β, *p*)	Steroid Use (β, *p*)
Reaction Time	0.09	−0.716, *p* = 0.095	0.157, *p* = 0.438	17.843, *p* = 0.173
Learning	0.393	−0.461 *, *p* < 0.001	−0.048, *p* = 0.751	1.568, *p* = 0.535
Working Memory	0.103	−0.201, *p* = 0.323	0.121, *p* = 0.518	13.122, *p* = 0.072
Spatial Memory	0.183	−0.217 *, *p* = 0.042	−0.093, *p* = 0.519	1.017, *p* = 0.739
Cognitive Flexibility	0.138	−0.257 *, *p* = 0.049	0.063, *p* = 0.656	0.427, *p* = 0.907

* *p* < 0.05; R^2^—coefficient of determination (model strength); β—regression coefficient; *p*—*p*-value (statistical significance). Source: Own elaboration/Author’s own work.

## Data Availability

The raw data supporting the conclusions of this article will be made available by the authors on request.

## Abbreviations

The following abbreviations are used in this manuscript:

ANAM	Automated Neuropsychological Assessment Metrics
RA	rheumatoid arthritis
VAS	Visual Analog Scale
BDI	Beck Depression Inventory
ESR	erythrocyte sedimentation rate
CNS	central nervous system
DMARDs	disease-modifying antirheumatic drugs
